# The Temporal Structure of Vertical Arm Movements

**DOI:** 10.1371/journal.pone.0022045

**Published:** 2011-07-12

**Authors:** Jérémie Gaveau, Charalambos Papaxanthis

**Affiliations:** 1 Unité 887 Motricité-Plasticité, INSERM, Dijon, France; 2 UFR STAPS, Université de Bourgogne, Dijon, France; Mayo Clinic College of Medicine, United States of America

## Abstract

The present study investigates how the CNS deals with the omnipresent force of gravity during arm motor planning. Previous studies have reported direction-dependent kinematic differences in the vertical plane; notably, acceleration duration was greater during a downward than an upward arm movement. Although the analysis of acceleration and deceleration phases has permitted to explore the integration of gravity force, further investigation is necessary to conclude whether feedforward or feedback control processes are at the origin of this incorporation. We considered that a more detailed analysis of the temporal features of vertical arm movements could provide additional information about gravity force integration into the motor planning. Eight subjects performed single joint vertical arm movements (45° rotation around the shoulder joint) in two opposite directions (upwards and downwards) and at three different speeds (slow, natural and fast). We calculated different parameters of hand acceleration profiles: movement duration (MD), duration to peak acceleration (D PA), duration from peak acceleration to peak velocity (D PA-PV), duration from peak velocity to peak deceleration (D PV-PD), duration from peak deceleration to the movement end (D PD-End), acceleration duration (AD), deceleration duration (DD), peak acceleration (PA), peak velocity (PV), and peak deceleration (PD). While movement durations and amplitudes were similar for upward and downward movements, the temporal structure of acceleration profiles differed between the two directions. More specifically, subjects performed upward movements faster than downward movements; these direction-dependent asymmetries appeared early in the movement (i.e., before PA) and lasted until the moment of PD. Additionally, PA and PV were greater for upward than downward movements. Movement speed also changed the temporal structure of acceleration profiles. The effect of speed and direction on the form of acceleration profiles is consistent with the premise that the CNS optimises motor commands with respect to both gravitational and inertial constraints.

## Introduction

Gravitational acceleration is omnipresent in our every-day life actions and plays an important role in several functions. For instance, its permanent action on the otolith organs of the vestibular system provides the Central Nervous System (CNS) with valuable information concerning spatial orientation, visual perception, and balance control [1], [2]. Gravity also influences movement elaboration. Previous studies have shown that the CNS takes advantage of gravity force in an optimal way during arm or whole body movements [3], [4], [5], [6]. Furthermore, it has been reported that the CNS uses an internal model of gravity to supplement sensory information when estimating time-to-contact with an approaching object [7], [8].

The performance of skilful movements requires the internal representation of the interaction of the body with the external world. The study of vertical arm movements offers an interesting paradigm to understand how the motor system deals with gravity force and what criteria are being applied during movement elaboration. For instance, if hand trajectories (path and/or velocity profile) are equivalent during upward and downward movements (i.e., under varying gravity effects), this may indicate a purely kinematic motor plan that accurately integrates gravity torques to preserve arm kinematics. On the other hand, significant changes in arm kinematics according to movement direction may indicate the existence of a dynamic planning process that takes advantage of the external forces acting on the limb to the detriment of the invariance of the hand trajectory. Indeed, previous studies have reported that kinematics differed between upward and downward movements, arguing thus in favor of a dynamic plan. Specifically, for various movements of equivalent duration and amplitude (pointing, reaching, drawing, and sit-stand-sit) acceleration duration is greater during downward than upward movements [3], [9], [10], [11]. In addition, parabolic-flight experiments revealed that exposure to new gravitational environments (micro and hyper-gravity) progressively modify this directional asymmetry, suggesting that gravity force is integrated into the central planning process [6], [12].

Although the analysis of acceleration and deceleration phases has permitted to widely explore the integration of gravity force during vertical arm movements, further investigation is necessary to conclude whether feedforward or feedback control processes are at the origin of this incorporation. In the case of a purely feedforward control mechanism, one would expect directional-dependent asymmetries to appear early, almost at the beginning of the movement. This may suggest an optimal control strategy based on the prediction of the mechanical effects of gravity on the moving segments. On the contrary, if directional-dependent asymmetries appear late during the motion, this may suggest a feedback control mechanism, which compensates gravity force on the basis of its effects on the moving segments. We considered that a more detailed analysis of the temporal patterns of vertical arm movements could provide additional information about the control mechanisms implied in the integration of gravity force into the motor command.

In the present study, healthy adults performed upward and downward vertical arm pointing movements at varying speeds (from 0.9 s to 0.35 s). In order to emphasize the effects of gravity, we simplified motion dynamics by imposing arm movements with one mechanical degree of freedom (rotation around the shoulder joint). During single-joint arm movements, inertia (i.e. the distribution of the arm mass around the shoulder) remains constant, and thus inertial torques are related only to joint acceleration. We consider that simplifying as much as possible the effects of inertia could permit us to better elucidate the role of gravity in the planning process of vertical movements. Our main findings showed that direction-dependent differences in acceleration profiles appeared early in movement execution (i.e., before peak acceleration) and remained until the moment of peak deceleration. This original result indicates differing motor intentions according to movement direction, and suggests that gravity force plays a vital role in movement elaboration.

## Materials and Methods

### Ethical statement

All participants gave their written informed consent prior to their inclusion in this study, which was carried out in accordance with legal requirements and international norms (Declaration of Helsinki, 1964), and approved by the Dijon Regional Ethics Committee.

### Participants

Eight right-handed healthy adults (all males, mean age = 24±3 years), without neuromuscular diseases and with normal or corrected-to-normal vision, participated in this study.

### Experimental device and protocol

**Figure 1:**
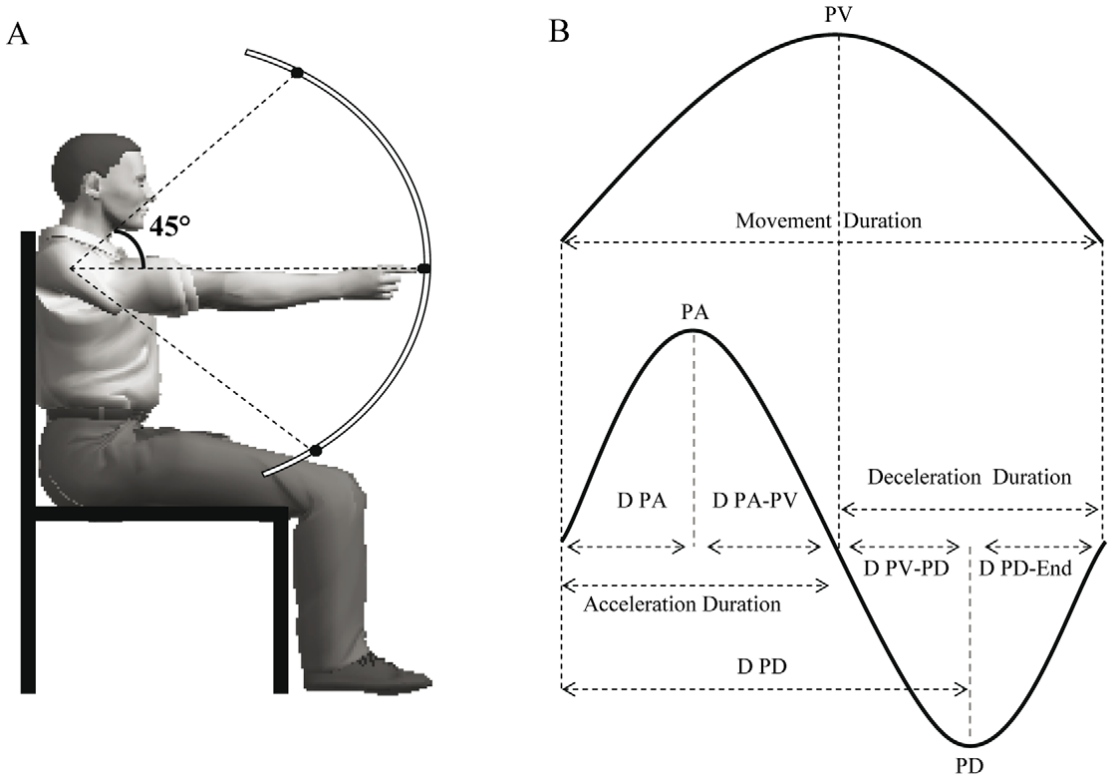
Experimental setup and data analysis. (A) Participants initial position and spatial location of the targets (right-side view). (B) We delimited movement duration by cutting velocity profiles with a 5% threshold of their peak velocity (PV). Several parameters were then determined on the corresponding acceleration profile: the peak acceleration (PA) and its time of apparition (D PA), the time between PA and PV (D PA-PV), the Acceleration Duration (time to PV), the deceleration peak (PD) and the time between PV and PD (D PV-PD); the time between PD and movement end (D PD-End); the deceleration duration (from PV to end).

Participants were comfortably seated on a chair with their trunk aligned in the vertical position. Three targets (plastic spheres, diameter of 1 cm, fixed on a steel semicircular bar) were centered on the participants' right shoulder (parasagittal plane) in a polar frame of reference at a distance equal to the length of their fully extended arm. The middle of the targets was aligned with the horizontal axis, while the other two targets were placed 45° upwards and 45° downwards (Fig. 1A). Participants were requested to perform visually-guided single-joint (rotation around the shoulder joint) upward and downward arm movements in the sagittal plane at three different speeds (slow, S; normal, N; fast, F). The required shoulder angular displacement (elevation angle), determined by the position of the targets, was 45° for all movement conditions. Before the experiment, participants had some practice trials to perform arm movements at approximately 0.8 s (Slow movements), 0.6 s (Natural movements) and 0.4 s (fast movements).

Slow, natural and fast movements were performed in a block design and were counterbalanced between the participants. Within each block, upward and downward movements were performed in a random order. Each participant accomplished ninety trials (15 trials per experimental condition). A short rest period (∼20 s) separated each trial, a rest of 2 min was given to participants after 15 consecutive pointing movements, and the three blocks were separated by a 5 min time interval in order to prevent muscle fatigue. One trial was carried out as follows: the participants visually placed their right arm in front of the middle target without touching it (in that initial position, the arm was aligned with the horizontal axis); after a variable period (between 2 s–3 s), the experimenter indicated the target to be pointed at (upward or downward); the participants, without any reaction time requirements, initiated their movement to this target. Participants were asked to produce uncorrected arm movement, without dedicating any particular attention to the final precision. They were also requested to maintain the final position of their arms (∼2 s) until they heard a verbal signal instructing them to relax their arm at their sides near the hip.

Arm movements were recorded using 4 TV-cameras (sampling frequency 120 Hz) of an optoelectronic system of motion analysis (Smart, B.T.S., Italy). Five reflective markers (1 cm in diameter) were placed on the shoulder (acromion), elbow (lateral epicondyle), wrist (in the middle of the wrist joint between the cubitus and radius styloid processes), hand (first metacarpophalangeal joint), and the nail of the index fingertip.

### Data analysis

After three-dimensional calibration (3-D), the spatial resolution for movement measurements in the present experiment was less than 1 mm. Data processing was performed by using custom software written in Matlab (Mathworks, Natick, MA). Recorded position signals in the three axes of the space (X, Y, Z) were low-pass filtered using a digital fifth-order Butterworth filter with zero-phase distortion (Matlab ‘butter’ and ‘filtfilt’ functions) at a cut-off frequency of 10 Hz. From position signals, we calculated velocity and acceleration profiles. The start and end of each trial was defined as the time that finger tangential velocity went above or fell below 5% of maximum velocity. A visual inspection of all trials revealed that velocity profiles were single-peaked and bell-shaped.

We computed angular displacements (elevation and azimuth) of each limb (upper arm, forearm, hand and finger) to ensure that subjects actually performed one degree of freedom vertical arm movements, i.e. rotating the shoulder joint in a parasagittal plane without any elbow, wrist or finger joint motion. Then, we calculated the subsequent kinematic parameters of the marker placed on the right index-fingertip (see Fig. 1B): movement duration (MD), duration to peak acceleration (D PA), duration from peak acceleration to peak velocity (D PA-PV), duration from peak velocity to peak deceleration (D PV-PD), duration from peak deceleration to the end of the movement (D PD-End), acceleration duration (AD), deceleration duration (DD), peak acceleration (PA), peak velocity (PV), and peak deceleration (PD). All these parameters can be modulated according to movement direction and movement speed, and therefore could provide information about the control process of vertical arm movements. We also calculated *invariant* parameters, such as the relative duration to peak acceleration (rD PA, defined as the ratio D PA/MD), the relative duration to peak velocity (rD PV, defined as the ratio AD/MD), and the relative duration to peak deceleration (rD PD, defined as the ratio D PD/MD). These parameters are called *invariant* because they could be independent of movement direction, speed and amplitude, thus providing information about the motor planning process of vertical arm movements.

To qualitatively illustrate similarities or dissimilarities in movement kinematics, finger tangential velocity and acceleration profiles were normalised in duration and amplitude for each trial and each participant. The normalization guarantees that velocity and acceleration profiles are independent of the distance travelled, the movement speed, and the movement duration.

Shoulder gravitational torques (SGT) were calculated using the following equations:
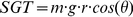
(1)where, *m* is the mass of the arm, *r* is the distance between the center of rotation of the shoulder joint and the centre of mass of the whole arm (upper arm+forearm+hand); *g* is the gravitational acceleration (9.81 m.s^−2^); *θ* represents the angle between the arm and the horizontal axis. The values for *m, and r* were calculated using the anthropometrical data given by Winter [13].

### Statistical analysis

Measures showed normal distribution (Shapiro-Wilk tests) and were submitted to a two-way analysis of variance (ANOVA). The factors examined were: *direction* (Upward versus Downward) and *speed* (S, N, and F). *Post-hoc* comparisons were performed by means of *Scheffé tests*. For all the statistical analyses the level of significance was fixed at *P*<0.05.

## Results

### General features

**Figure 2:**
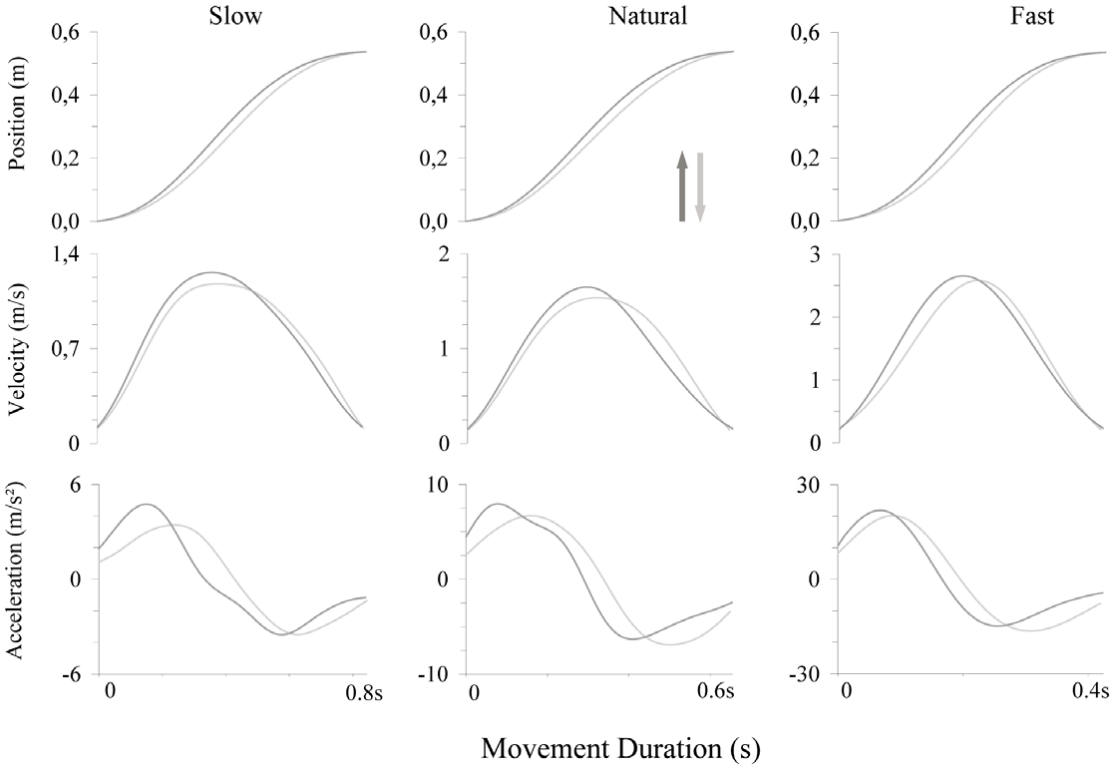
Arm Kinematics. Typical profiles of hand position, velocity and acceleration for all experimental conditions. Vertical arrows indicate movement directions.

Fig. 2 illustrates typical kinematic features of vertical arm movements. Velocity profiles were singled-peaked and acceleration profiles double-peaked with one peak during the acceleration phase and one peak during the deceleration phase. All participants performed arm movements by mobilizing only the shoulder joint (no rotation was observed at the other joints) and without deviating from the sagittal plane; for all trials (n = 720), shoulder azimuth angle was inferior to 1°. Furthermore, arm movements were accurate overall (average shoulder elevation: 43.7±3.5°) and showed little variability (on average: 1.3°±0.5°). In our experiment, the main external constraint applied on the moving arm was gravity force. The average SGT before movement onset was 13.15±1.22 N⋅m. Due to the symmetric location of upward and downward targets with respect to the horizontal axis, SGT similarly decreased during arm movements in both directions (on average: 3.94±0.42 N⋅m).

### Movement duration

**Figure 3:**
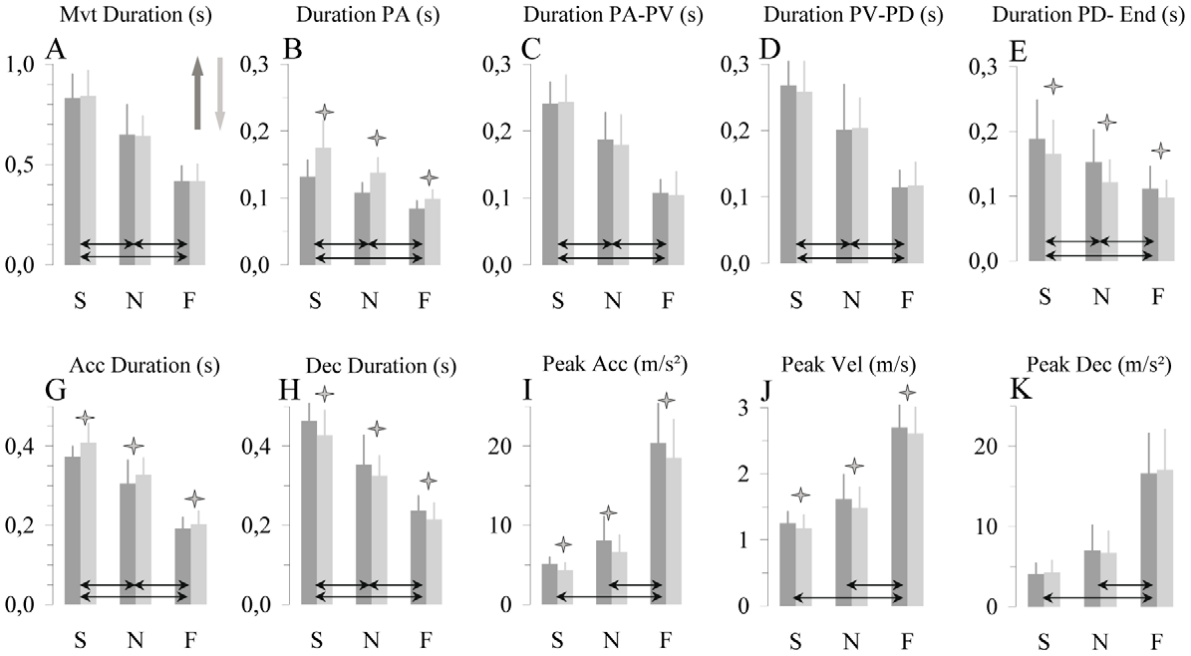
Temporal parameters of arm movements. Averaged (n = 8) values of non normalized temporal parameters (± SD) for upward and downward arm movements performed at slow (S), natural (N) and fast (F) speeds. (A) movement duration, (B) time of apparition of peak acceleration, (C) duration between peak acceleration and peak velocity, (D) duration between peak velocity and peak deceleration, (E) duration between peak deceleration and movement end, (G) acceleration duration, (H) deceleration duration, (I) peak acceleration, (J) peak velocity, (K) peak deceleration. Vertical arrows indicate movement directions. Stars indicate differences between directions and horizontal black arrows differences between speeds.

Average movement durations were 0.84 s, 0.64 s and 0.42 s for slow, natural and fast movements, respectively. As required by the experimental instructions, movement duration (see Fig. 3A) significantly varied with movement speed (*speed* effect; F_2,14 = _31.15, P<0.0001). Post-hoc comparisons showed that durations of slow, natural and fast movements significantly differed between them (P<0.001, for all comparisons). However, within each movement speed, upward and downward movements had similar durations (F_1,7 = _0.15, P>0.7). Interaction between *speed* and *direction* did not reach significance (F_2,14 = _0.03, P>0.9).

### Duration to peak acceleration (D PA)

D PA (see Fig. 3B) significantly decreased when movement speed increased (F_2,14 = _17.43, P<0.0001). Post-hoc comparisons showed that D PA significantly differed between slow, natural and fast movements (P<0.05, for all comparisons). Interestingly, D PA was shorter for upward than downward movements for all movement speeds (*direction* effect; F_1,7 = _17.43, P<0.005). The difference in D PA between upward and downward directions tended to decrease when movement speed increased; however, the interaction between *speed* and *direction* was not significant (F_2,14 = _3.71, P>0.05).

### Duration from peak acceleration to peak velocity (D PA-PV)

D PA-PV (see Fig. 3C) was also modulated by movement speed (F_2,14 = _24.83, P<0.0001). D PA-PV of slow, natural and fast movements significantly decreased when movement *speed* increased (P<0.05, for all *post-hoc* comparisons). However, D PA-PV was similar for upward and downward movements (F_1,7 = _0.61, P>0.4) for all movement speeds. The interaction between *speed* and *direction* was not significant (F_2,14 = _0.71, P>0.5).

### Duration from peak velocity to peak deceleration (D PV-PD)

Movement speed significantly influenced D PV-PD (see Fig. 3D), which decreased when movement speed increased (F_2,14 = _16.24, P<0.0001). *Post-hoc* comparisons showed that D PV-PD significantly differed between slow, natural and fast movements (P<0.05). Direction did not influence D PV-PD (F_1,7 = _0.55, P>0.4), which was similar for upward and downward movements. The interaction between *speed* and *direction* was not significant (F_2,14 = _0.55, P>0.5).

### Duration from peak deceleration to the end of the movement (D PD-End)

D PD-End (see Fig. 3E) was significantly influenced by movement speed (F_2,14 = _5.90, P = 0.01) and by movement direction (F_1,7 = _8.45, P = 0.02). D PD-End significantly decreased when movement *speed* increased (P<0.05, for all *post-hoc* comparisons). Interestingly, D PD-End was shorter for downward compared to upward movements. This direction-dependent modulation was opposite to that observed for D PA. The interaction between *speed* and *direction* was not significant (F_2,14 = _0.57, P>0.55).

### Acceleration duration (AD) and deceleration duration (DD)

From the above-described data, it appears that AD and DD differed between upward and downward movements (see Fig. 3 G and H). Upward movements had shorter AD (F_1,7 = _11.29, P = 0.01) and longer DD (F_1,7 = _9.51, P = 0.02) than downward movements. Furthermore, upward movements were more asymmetric than downward movements. The average (n = 8) ratio of AD/DD for upward movements was 0.83, while for downward movements it was 0.97; the ratio 1 indicates perfect symmetry (AD = DD). Both AD and DD were modulated accordingly to movement speed (F_2,14 = _43.66, P<0.0001 for AD ; F_2,14 = _21.25, P<0.0001 for DD). Post-hoc comparisons showed that AD and DD of slow, natural and fast movements significantly decreased when movement *speed* increased (P<0.05, for all comparisons).

### Peak acceleration, peak velocity, and peak deceleration

Fig. 3I–K shows average values (± SD) of PA, PV, and PD. It is noticeable that PA (F_1,7 = _9.59, P = 0.02) and PV (F_1,7 = _8.32, P = 0.02) were greater for upward than downward movements, while PD was equivalent for the two movement directions (F_1,7 = _0.07, P>0.7). Furthermore, the average values of these kinematic parameters increased according to movement speed (for all comparisons, P<0.0001). Post-hoc analysis revealed for all parameters significant differences between S and F (P<0.01), and N and F (P<0.01) movements, but not between S and N (P>0.05).

All the above-described kinematic features are displayed in the Fig. 2.

### Normalized velocity and acceleration profiles

**Figure 4:**
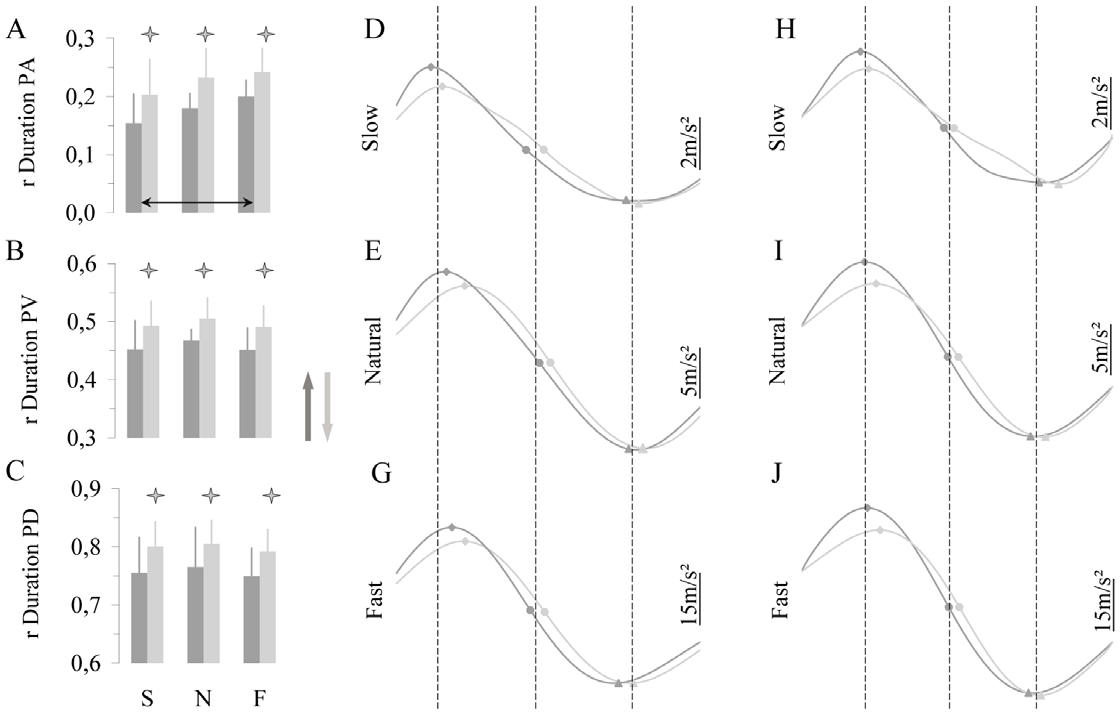
Normalized temporal parameters and normalized acceleration profiles of arm movements. Left column: averaged (n = 8) values (± SD) of (A) relative duration to peak acceleration, (B) relative duration to peak velocity, (C) relative duration to peak deceleration; slow (S), natural (N) and fast (F) speeds. Middle column: normalized (in duration) and averaged (8 subjects) acceleration profiles in which movement start and end were determined with the ‘*percentage threshold method*’. (D) Slow movements, (E) natural movements, (G) fast movements. Right column: normalized (in duration) and averaged (n = 15 trials) acceleration profiles from a representative subject in which movement start and end were determined by a ‘*fix value threshold method*’ (1 m/s^2^, 3 m/s^2^ and 10 m/s^2^ for slow, natural and fast speeds respectively). (H) Slow movements, (I) natural movements, (J) fast movements. Dashed vertical lines are depicted to compare profiles between speed conditions. Vertical arrows indicate movement directions. Stars indicate differences between directions and horizontal black arrows differences between speeds.

Fig. 4A–C shows average values (± SD) of the relative duration to PA, PV and PD. These parameters are clearly modulated according to movement direction (rD PA, F_1,7 = _18.34, P<0.01; rD PV, F_1,7 = _81.18, P<0.0001; rD PD, F_1,7 = _12.38, P<0.001). There was also an effect of movement speed, but only for rD PA (F_2,14 = _5.82, P<0.01). Post-hoc comparisons revealed differences between slow and fast movements (P = 0.02), but not between slow and natural movements (P = 0.13) or between natural and fast movements (P = 0.51). Speed did not affected rD PV and rD PD (for all comparisons, P>0.05). Fig. 4 D–G qualitatively illustrates the dissimilarities of acceleration profiles according to movement direction and their similarities according to movement speed.

### Control analysis

Many of the previous studies had determined movement start and end by using threshold percentages of peak velocity [3], [4], [6], [11], [12], [14], [15], [16]. Here, we used a threshold value of 5%. However, as our results showed an effect of movement direction on peak velocity, we wondered whether a threshold percentage could influence our temporal parameters. To insure that our findings were independent from our *percentage method*, we performed a new analysis on acceleration profiles by using fixed values. Precisely, we chose minimum fix values to automatically determine the start and the end of the movement, i.e. 1 m/s^2^, 3 m/s^2^ and 10 m/s^2^ for slow, natural and fast speeds respectively. We observed very similar results irrespective of the method used for cutting velocity profiles (see Fig. 4 H–J for an illustrative example).

## Discussion

In the current study, we analyzed acceleration profiles of vertical arm movements to explain how the brain integrates gravitoinertial forces into motor planning. Precisely, we investigated whether directional-dependent kinematic asymmetries, that were previously reported for arm movements performed in the sagittal plane, were due to feedforward or feedback control processes. We found that upward versus downward kinematic asymmetries emerged early (i.e., before peak acceleration) during movement execution. Additionally, we observed that temporal organization of vertical arm movements varied with speed. These findings denote specific motor intentions according to both movement direction and speed.

### Effects of movement direction

Our results revealed that direction is a discriminative factor in the production of vertical arm movements. We found that while movement durations and amplitudes were similar for upward and downward movements, the internal temporal structure of acceleration profiles significantly differed between the two directions. Specifically, subjects executed upward movements faster than downwards until the moment of peak deceleration; that is, duration to peak acceleration, duration to peak velocity, and duration to peak deceleration were all shorter for upward than downward movements. Additionally, the values of peak acceleration and peak velocity were greater for upward than downward movements. These findings confirm and extend previous observations that revealed similar direction-dependent asymmetries during the motion of the whole body [3], the upper limb [9], [10], [11], [16], and the lower limb [17], [18]. Notably, this asymmetry is a specific feature of vertical movements, because it was not observed for horizontal movements [11], [15], [19], [20].

It is of interest that direction-dependent kinematic asymmetries appeared early in movement production (see Fig. 2 and Fig. 4D–J). They emerged, specifically, between movement initiation and peak acceleration and remained unchanged until the end of the movement. The shortest feedback corrections for arm movement were observed around 150 ms on kinematics and 100 ms on EMG patterns [21], [22], [23], [24], [25]. Because kinematic asymmetries appeared at the beginning of the movement (see Fig. 4H–J), and notably before 100 ms for fast movements (see Fig. 2 and 3B), one could argue in favor of a purely feedforward process for the control of vertical arm movements. This original finding denotes differing motor intentions according to movement direction, and suggests that gravity force plays an important role in movement elaboration. The motor plan is related to the choice of a motor pattern among the several possibilities that could satisfy the goal of the movement. Its content is accessible by examining movement characteristics (i.e., normalized parameters that are not directly tuned by task constraints) that remain invariant under differing experimental conditions. In our study, such independent features were the relative duration to peak acceleration (rD PA), the relative duration to peak velocity (rD PV), and the relative duration to peak deceleration (rD PD). If the CNS has a general kinematic plan, these normalized parameters should remain stable across movement directions. Such a kinematic strategy has been proposed for horizontal arm movements [11], [20] and can be predicted by optimization models such as the minimum jerk model [26]. However, in the case of vertical arm movements, rather than producing similar upward and downward acceleration profiles by counterbalancing gravitational torques with muscular force, the CNS uses gravity force to brake (upward direction) or initiate (downward direction) arm motion. These findings denote a dynamic, rather than purely kinematic, planning process that integrates gravity force. The observation that acceleration and velocity peaks were larger during upward than downward movements (i.e., a disproportional motor command between the two directions) shows an intention to overcome gravity torque. Precisely, this strategy releases time for decelerating upward movements (longer durations during upward than downward movements) and makes possible the use of gravity torque during deceleration. Conversely, downward movements presented longer acceleration and shorter deceleration durations; this strategy releases time for gravity force to accelerate the arm downwards. The analysis of muscle activation patterns during vertical arm movements also supports this assumption [16], [27]. Such a strategy is highly appealing, notably due to its great compatibility with optimal and stochastic motor control theories, and denotes an efficient manner to minimize the energetic cost of movements performed in the vertical plane [4], [6]. Additionally, because this strategy minimizes muscular work, and thus motor command, it should also reduce signal dependent noise [28], [29], [30]. In this theoretical context, the finding that directional asymmetries were entirely produced during the first (D PA) and compensated during the last (D PD-End) phase of the movement logically argues for a maximization of gravitational effects as a potential resource for the motion of the arm.

### Effects of movement speed

Our findings showed that acceleration profiles did not remain invariant with movement speed. Precisely, we observed that the relative duration to peak acceleration changed with movement speed (see Fig. 4A, and D–J). Note that a basic scaling strategy should produce equivalent acceleration profiles for slow, natural, and fast movements. For instance, speed-invariant strategy was previously proposed for arm postures [31] and velocity profiles [14] during 3D arm movements. We think that these differences were due to the specific muscle activation patterns of vertical arm movements which varied according to movement speed. We have previously reported that when movement speed decreased subjects guided vertical arm movements by increasing or decreasing muscular force of the flexor muscles only; precisely, concentric contraction for upward movements and eccentric contraction for downward movements [16]. This EMG pattern suggests that the CNS exploits the gravitational force [32], which progressively replaces muscular force and optimises the overall muscle activation when movement speed decreases. Optimal control models that take into account gravity effect upon vertical arm movements [4], [6] might reproduce such speed effect because of increasing energetic costs. In this way, we recently reported that the CNS takes advantage of the gravitational acceleration to reduce muscle activations associated with both upward and downward arm movements [27].

In our study, speed-dependent effects on the form of acceleration profiles are consistent with the premise that the CNS optimises motor command with respect to both gravitational and inertial constraints. We recently showed that the CNS differently adapts arm kinematics in response to gravitational and inertial constraints [33]. If gravity and inertia were not included in the planning process, arm kinematics should not, *a priori*, have changed with both direction and speed in the current study. Specifically, if only gravity was the relevant parameter for motor planning, we should observe direction-dependent, but not speed-dependent, kinematic modulations, and vice versa. Current results could be explained by theoretical models minimizing energetic type costs [4], [6]. Logically, minimizing energy expenditure implies movement parameters that have been adapted to both gravitational and inertial constraints of the task [33], [34]. Gravity torques (which are position-dependent) exert a major influence on motion dynamics at relatively slow speeds, while inertial torque (which are velocity-dependent) exert a major influence at relatively fast speeds. Recent studies have proposed that the CNS independently controls spatial and temporal components of arm movements [35], [36], [37]. It appears, thus, plausible that the CNS could independently tune movements in response to gravitational and inertial properties of the system to be controlled.

Overall, our findings suggest that the brain, by integrating gravity into the motor planning process, has developed a specific motor law for the performance of vertical arm movements. Direction-dependent asymmetries observed in our experiments could be the outcome of a control strategy that optimizes gravity force. Indeed, the minimum absolute work model [4] predicts the experimentally recorded kinematics of upward and downward movements. Current results are thus in accordance with the general consensus that the brain optimally integrates external force into the planning process in order to reach the goal of a motor action at a minimum cost [38], [39].

### Neurophysiological evidences

Several neurophysiological findings are compatible with behavioral evidence arguing in favor of a central integration of gravitational force. Arm movement direction and velocity, as well as external forces, are represented in several brain areas, including the motor, premotor and parietal cortices where neuronal populations encode the direction of the arm movement in space [40], [41], [42], [43]. Furthermore, the direction of an external force, like the direction of gravity, can be controlled independently of its magnitude and this directional signal is especially prominent in the motor cortex. For instance, during isometric force production against external forces of different directions and magnitudes, cell activities in the motor cortex of monkeys were modulated by the direction, the magnitude, or both the direction and magnitude of the external force [see 42 for a review], [44]. Additionally, recent neurophysiological data reported that neural processing between the premotor and primary motor cortices contributes to the sensorimotor transformations between extrinsic (direction) and intrinsic (arm posture, muscle activity) representations of limb movement [45]. In particular, during isometric motor tasks the discharge rates of cells in the primary motor cortex were significantly affected by both hand location and the direction of external static forces [46], [47].

In conclusion, our study suggests that the human motor system internally represents limb and environmental dynamics. The kinematic analysis of vertical arm movements appears to be a reliable and powerful tool to investigate motor planning and optimal processes in motor control. Such a simple paradigm could be very useful in evaluating motor impairment related to ageing or neurodegenerative diseases and to further investigate the adaptation process to new gravitoinertial environments. New investigations, exploring the electromyographic patterns of vertical arm movements, could contribute to a better understanding of the strategy developed by the CNS to control vertical movements.
